# The state of UK palliative care – time for change at scale and pace

**DOI:** 10.1016/j.fhj.2026.100508

**Published:** 2026-03-27

**Authors:** Paul Paes, Suzanne Kite

**Affiliations:** Association for Palliative Medicine of Great Britain and Ireland, Lancaster Court, 8 Barnes Wallis Road, Fareham, PO15 5TU, England

**Keywords:** Palliative care, Quality of life, Transformation, Funding

## Abstract

After 60 years of development, palliative care services are fully embedded in local communities, raising awareness, delivering care and often also relying on local fundraising. Specialist palliative care teams are integrated within primary and community care services, as well as acute hospitals. Palliative care is shown to be cost effective, improving patient experience and reducing acute hospital bed days, saving money for the health service as long as patients access services early enough to make a difference. Unfortunately across the country, services are patchy with significant inequalities due to a lack of universal funding, clear outcomes and accountability. The likely implementation of Assisted Dying Bills brings to a head the likelihood that services that assist people to die will be available on the NHS, but not those services assisting people to live. The specialty is ready to embrace the shifts to community-based, preventative care driven through technological change. Palliative care has always been innovative and agile, focused on problem solving, relationship building and personalised care. Political leadership now could truly unlock the care of those towards the end of their life. A fully funded palliative care service with a clear national service framework backed by clear outcome measures could transform the quality of palliative and end of life care, offering patients real choice and true ‘cradle to grave’ care. With that in place, palliative care is ready to respond at a scale and pace not previously seen.

## Graphical abstract

**Figure:**
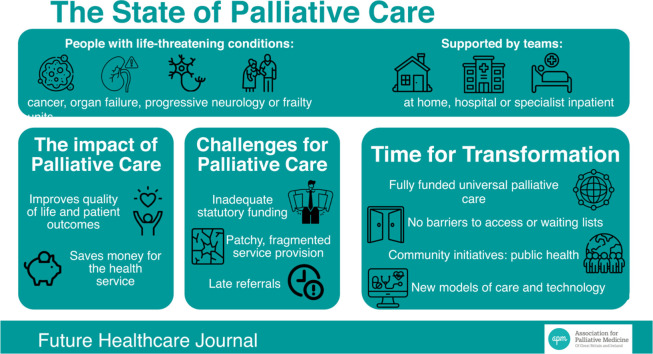

Palliative care in the UK is at a crossroads. The evidence base for the effectiveness of services has grown significantly yet the specialty is challenged by a funding crisis and the potential introduction of Assisted Dying Bills. This article will explore the nature of palliative care, the development of services, successes and challenges before looking to the future and the scale of change required.

## What is palliative care?

The World Health Organization (WHO) defines palliative care as ‘an approach that improves the quality of life of patients and their families who are facing problems associated with life-threatening illness. It prevents and relieves suffering through the early identification, impeccable assessment and treatment of pain and other problems, whether physical, psychosocial, or spiritual’. This approach applies both early in the course of an illness alongside other life-prolonging therapies, and later to help people live as actively as possible until death.^1^

**Fig. 1:**
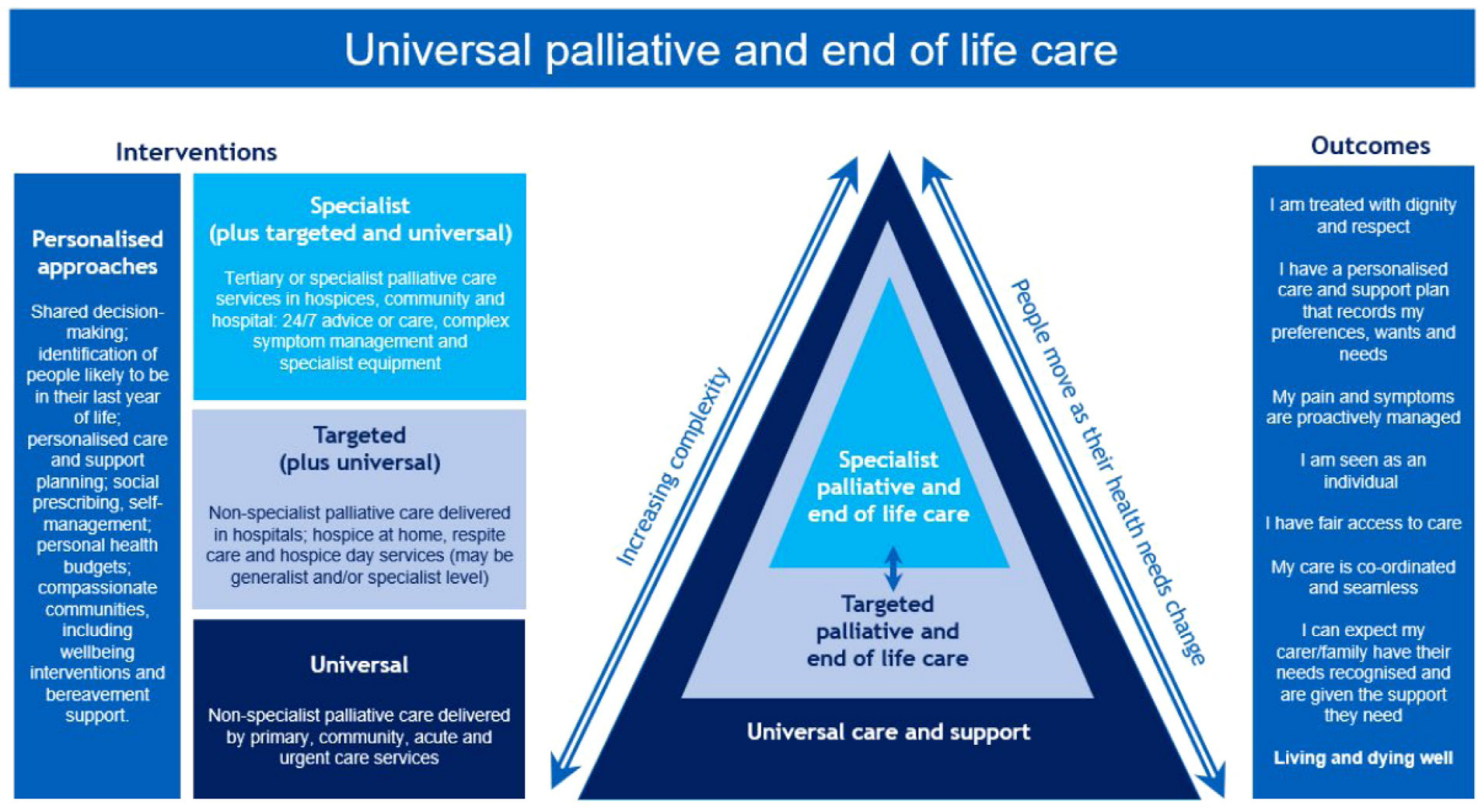
Relationship between services. Source: NHS England, licensed under the Open Government Licence v3.0.^3^

Palliative and end-of-life care is practised by a range of different professionals, many delivering palliative care alongside other clinical services, coupled with specialists who have undertaken extra training working exclusively in this area.^2^ The diagram below sets out this model with ‘universal’ palliative care delivered by all clinicians, ‘targeted’ non-specialist palliative care and specialist palliative care (Fig. 1).^3^

Specialist palliative care (SPC) services look after patients with a range of malignant and non-malignant conditions, though cancer continues to be the most common. Alongside SPC, supportive care in cancer focuses on ‘the prevention and management of the adverse effects of cancer and its treatment. This includes management of physical and psychological symptoms and side effects across the continuum of the cancer experience from diagnosis, through anticancer treatment, to post-treatment care. Supportive care aims to improve the quality of rehabilitation, secondary cancer prevention, survivorship, and end-of-life care’.^4^

The scope of SPC extends far beyond direct clinical care. Education and training are central. Patient education includes public health campaigns to encourage people to talk about what is happening to them, their hopes and fears as well as enabling them to prioritise their wishes. Education of primary, community, acute and urgent care services enables universal end-of-life care to be delivered. E-ELCA (End of Life Care for All) is a national e-learning programme designed to upskill all of those who work in end-of-life care.^5^ Quality improvement and service evaluation are integral to service delivery, for example the development of Electronic Palliative Care Co-ordination Systems (EPaCCS) or the National Audit of Care at the End of Life: Dying in Hospitals.^6^ Finally, research has been crucial in developing the evidence base for palliative care and evaluating new treatments, interventions and models of care.

## The development of palliative care

Sixty years ago the first hospice opened, followed by community and then hospital SPC teams. Since then, services have become fully embedded in local communities, raising awareness, delivering care and often relying on local fundraising. Similarly, SPC teams are integrated within primary and community care services, and acute hospital services.

Palliative care is taught in undergraduate and postgraduate curriculums of most health disciplines, and is a source of satisfaction for many health professionals. A very small specialty, with a few nurses and even fewer doctors, has grown into a fully developed multiprofessional service with individual disciplines developing their own expertise, evidence and guidance. NICE recommended in 2004 that SPC teams should provide:

• assessment, advice and care for patients in community and hospital settings 7 days a week, backed up by telephone advice at all times
• specialist inpatient facilities (hospices or hospitals) for patients with complex problems who would benefit from the continuous support of a multidisciplinary SPC team with full medical cover. Multiprofessional teams require palliative medicine consultants, nurse specialists, physiotherapists, occupational therapists, dietitians, pharmacists, social workers, chaplains/spiritual care givers and administrators, supported by a range of important services.^7^

By 2024 there were an estimated 791 palliative medicine consultants in post (569 whole-time equivalent (WTE)) in the UK with another 10% of vacancies, complemented by 350 WTE fellow and SAS (specialist, associate specialist and specialty) doctor posts.^8^ Currently, training bottlenecks, and a growth in posts and retirements, means that the supply of new consultants is not meeting demand. In the UK, there are approximately 0.85 WTE consultants in palliative medicine per 100,000 population, whereas in two comparator countries, Australia and Ireland, the corresponding national recommendation is two WTE consultants.^9^

Previously, doctors entered SPC training with a wide variety of backgrounds, particularly general practice, anaesthetics and surgery. This gave a broad range of applicants and a diversity of skills. Evolution of training programmes, particularly with Shape of Training, has created new opportunities, but closed off SPC to others. Integrating SPC with general medicine broadens the skillset for specialists to support patients with palliative care needs arising from long-term conditions and frailty, while creating more varied joint posts in general and acute medicine. Options to gain dual accreditation in primary care and palliative medicine are being explored and would significantly support care at home.

## Palliative care successes

The Commission on Palliative and End of Life care summarised the benefits of SPC from Cochrane reviews: it enhances quality of life, emotional wellbeing and symptom management for individuals in community and hospital settings.^10^ It improves outcomes and in some situations, improves survival.^11^ Palliative care is cost effective, improving patient experience and reducing bed days, which reduces healthcare costs considerably.^12^ Patients need to access palliative care early enough to make a difference; SPC services are most effective when started at least 3 months before death.^13^ Three key areas with the greatest weight of evidence of effectiveness and overall potential cost savings are:

• earlier identification of palliative care needs in hospitals, and earlier referral for SPC during admission
• equitable palliative care and end-of-life care, delivered by generalist- and specialist-integrated neighbourhood teams
• robust out-of-hours care with prompt home visits for crisis care and dedicated palliative care telephone support lines in all areas, staffed by professionals with experience and training in palliative care.^14^

The number of community, hospital and inpatient services has expanded and often become integral to the local health system.

## Palliative care challenges

However, there is considerable unmet need, with patients often not seen or referred too late to benefit from palliative care interventions.^13^^,^^15^ The palliative care needs of the population are growing as people live longer with multiple long-term conditions and the number of deaths each year is increasing.^16^ About 40% of hospitals lack 7-day SPC services^17^ and many areas in the UK do not provide 24/7 telephone advice. The specialty currently faces an existential threat, triggered by dual challenges: funding and assisted dying. Universal NHS funding for palliative care services has not been achieved despite multiple strategy documents, NICE guidance and positive economic evaluations. The commissioning of SPC is hampered by the lack of a funding currency and national outcomes to drive improvement. Reliance on charity is unique to end-of-life care services. Unfortunately, the increased cost of living has decimated charitable giving, resulting in hospice closures or services reductions. Primary, social and community care, equally integral to high-quality end-of-life care, are all struggling to meet clinical demand.

**Fig. 2:**
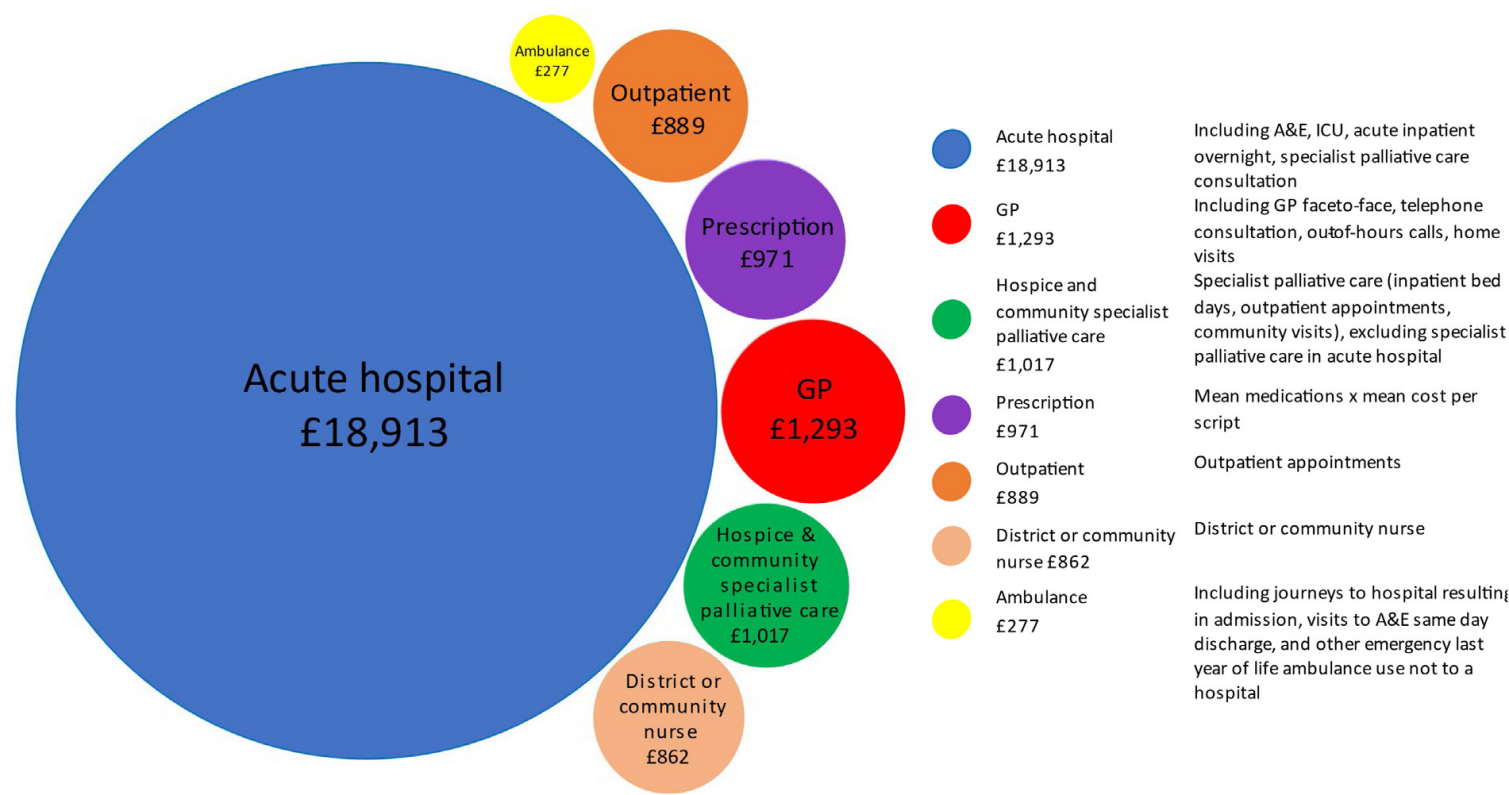
Composition of total formal healthcare costs per decedent in the last year of life, based on 2017 costs and adopting standard inflation to 2024 costs. (Bubble area represents the relative proportion.) Permission to publish received from the lead author of report.^14^

Currently, 8% of the NHS budget is spent on the 1% of people who die each year, costing about £24,222 per patient. Of this only 6% is spent on SPC services, and hospital SPC costs are unknown (Fig. 2).^14^

A small shift from reactive emergency care to proactive planned care would enable greater investment in vital community services, while saving money. The UK has struggled to come up with a funding mechanism, outcome measures or service frameworks that can drive palliative care service development. For this, the UK now has to look internationally to Australia or the Nordic countries where universal palliative care has structures and funding to drive improvements.

The likely implementation of Assisted Dying Bills challenges many working in end-of-life care. Associated debates have crystallised the funding inequalities, with Parliaments voting for fully funded assisted dying services while not voting for fully funded palliative care. It is unthinkable that the UK may shortly be in a position where the state will assist people to die, but not to live until they die. ‘Assisted living’ will continue to remain largely dependent on charity. Real choice requires patients to have genuine high-quality options to choose from – access to social care, primary and community care, secondary care and particularly SPC must match that of assisted dying. Both can co-exist, but only where there is a full commitment to universal palliative care.

## The evolving role of specialist palliative care services

Palliative care services have pioneered many of the shifts set out in the NHS strategy:

***Treatment to prevention:*** Pioneering approaches such as the Compassionate Communities programme support community resilience.^18^ Population approaches to risk stratification and use of palliative care registers guide proactive care delivery. Supportive care models focus on preventative work.
***Hospital to community***: Community teams based around clusters of GP practices identify patients with palliative care needs, use electronic systems to monitor care and regularly assess needs. Increasingly hospital SPC teams are working in the emergency departments of hospitals, triaging patients to community settings. The shift from hospital to community in palliative care is proven in terms of cost and effectiveness, unlike other areas.^19^
***Analogue to digital:*** Digital systems enable integrated clinical records and coordinated personalised care plans. Artificial intelligence can triage the needs of a large population. Improved functionality enables connection with patients in multiple ways, mindful of the association of poor health outcomes and digital literacy, alongside connectivity issues.

These initiatives have changed where people are dying in the last decade,^20^ notably at home (28.4% in 2023 compared to 23% in 2014) and in hospital (42.8% in 2023 compared to 47.2% in 2014).

## What will be needed in the future?

The Royal College of Physicians called for a cultural shift away from the focus on curative treatments in hospital when many of the patients have progressive life-limiting conditions following a predictable course and are most likely to die in hospital.^21^ Open supportive conversations can recognise this and lead to a change in treatment approach.^22^ Transition points, such as an episode of unplanned care, provide opportunities to discuss holistic needs, future risk of admission and involve appropriate services.^23^

Patient complexity is increasing as patients live with multiple comorbidities and frailty. SPC does not have exclusivity over a disease process, age range or organ, but works collegiately alongside other professionals. Areas such as early supportive care blur the boundaries of prognosis and discipline. Complex decision making, patient empowerment, managing and living with uncertainty, and building systems sit comfortably with primary, older person’s and palliative care. Community neighbourhood teams will need to work as single teams with specialties integrating in new ways to avoid burdensome duplication. Patients will need to identify with one neighbourhood team that can deal with most of their health needs. Specialists will have to be flexible in taking the lead in some situations and stepping back in others. While embracing the technology, remaining personal and responsive to patient vulnerability will continue to be at the heart of our practice.

Hospital models will continue to require responsiveness and innovation to identify patients early, intervene and divert into planned community pathways or inpatient units. Shape of Training enables palliative medicine specialists to look after patients with different conditions more confidently and develop more portfolio careers. The further growth of hospital SPC units seems likely as integrated patient flow systems. SPC inpatient units need to be responsive, deliver high-quality patient experience, clear outcomes and show value for money. That will require convergence and consistency. Innovation will ensure that the significant increase in demand for education and training can be accommodated so that end-of-life care remains everyone’s responsibility.

### Time for transformation

For all palliative care services, responsiveness, patient experience and quality are crucial: early access with no barriers, no hand-offs and no waiting lists. Commitment to data, quality improvement and technology must drive all change. Palliative care services need to be outward looking, agile and operate at sufficient scale to enable full 24/7 access. In turn, other disciplines must embrace the realities of patients deteriorating, open up dialogue, refer early and eliminate gatekeeping. Finally, if the NHS is finally to deliver ‘cradle to grave’ care, palliative care services must be fully funded by the NHS in all parts of the country. Any new strategies or service frameworks need to be effective this time, with incentives to force change and penalties for failing to deliver. New models, new patient groups, new technology but still services committed to creative problem solving, relationship building and personalised care. Plus ça change, plus c’est la même chose? Yes, but needed at a pace and scale that has not been seen before.

## Funding

This article did not receive any grant from funding agencies in the public, commercial or not-for-profit sectors.

## CRediT authorship contribution statement

**Paul Paes:** Writing – review & editing, Writing – original draft, Conceptualization. **Suzanne Kite:** Writing – review & editing, Writing – original draft, Conceptualization.

## Declaration of competing interest

The authors declare that they have no known competing financial interests or personal relationships that could have appeared to influence the work reported in this paper.
